# Associations between Oral Health and Cannabis Use among Adolescents and Young Adults: Implications for Orthodontists

**DOI:** 10.3390/ijerph192215261

**Published:** 2022-11-18

**Authors:** Austin Le, Edmund Khoo, Joseph J. Palamar

**Affiliations:** 1 Department of Population Health, New York University Grossman School of Medicine, New York, NY 10016, USA; 2 Department of Orthodontics and Dentofacial Orthopedics, New York University College of Dentistry, New York, NY 10010, USA; 3 Department of Orthodontics and Oral Facial Genetics, Indiana University School of Dentistry, Indianapolis, IN 46202, USA; 4 Eastman Institute of Oral Health, University of Rochester, Rochester, NY 14620, USA

**Keywords:** cannabis, drug use, oral health, periodontitis, caries, orthodontist

## Abstract

Cannabis use is prevalent among adolescents and young adults in the US. Virtually all modes of cannabis consumption involve the oral cavity, and previous studies have linked cannabis use with poorer oral health. We sought to identify associations between cannabis use and various oral health outcomes and behaviors among individuals 12–25 years of age, and to discuss implications for orthodontists who largely interact with this age group over an extended period of treatment time. We examined data from patient electronic health records (N = 14,657) obtained between 2015 and 2021. Associations between lifetime and current self-reported cannabis use and several oral health outcomes or related behaviors that reflect periodontal health, caries status, oral lesions, and physical integrity of tooth structure and restorations were examined in a bivariable and multivariable manner, controlling for patient age, sex, and self-reported tobacco and alcohol use. Reporting lifetime cannabis use was associated with higher risk for having oral lesions (aPR = 1.41, 95% CI: 1.07–1.85), bruxism (aPR = 1.31, 95% CI: 1.09–1.58), and frequent consumption of sugary beverages and snacks (aPR = 1.27, 95% CI: 1.12–1.41). Reporting current cannabis use was associated with higher risk for oral lesions (aPR = 1.45, 95% CI: 1.03–2.06) and frequent consumption of sugary beverages and snacks (aPR = 1.26, 95% CI: 1.07–1.48). Cannabis users aged 12–25 are at increased risk for bruxism, oral lesions, and frequent consumption of sugary beverages and snacks. Orthodontists and other dental professionals should probe for drug use and be cognizant of increased risk for oral health problems in patients that report actively using cannabis.

## 1. Introduction

Cannabis remains the most prevalent psychoactive drug in the United States (US) that is illegal to possess at the federal level, and the prevalence of use, as well as cannabis use disorder, has steadily risen over the years [1,2,3,4,5]. Recreational cannabis use was legalized in New York State as of 31 March 2021 [6]. As the legal landscape in the US continues to shift towards decriminalizing or legalizing medical and recreational use [7], there is growing concern that younger age groups may be more likely to use cannabis and experience adverse effects on health.

Indeed, recent increases in mainstream media coverage of adverse effects of cannabis use among adolescents and young adults has reignited concerns over problems such as psychosis and cannabis hyperemesis [8,9,10,11,12], which are known to be associated with frequent or chronic use [13,14]. Furthermore, a recent study on the effects of cannabis legalization in the US found that cannabis legalization led to significantly higher risk of self-reported past-year cannabis use among a sample of 10–20 year old youth [15]. In 2019, an estimated 44% of 12th graders in the US had used cannabis in their lifetime, while 36% had used in the past year [16]. Researchers have suggested that increased risk for use may be attributable to an increase in perceived ease of access to cannabis products post-legalization [17], while the harmful effects may be due to the increase in the average concentration of tetrahydrocannabinol (THC), the main psychotropic agent in cannabis [18]. For example, a systematic review found that the average THC concentration in herbal cannabis has increased by approximately 0.29% each year between 1970 and 2017, and by over 0.5% annually in cannabis resins [19]. Whereas the average THC concentration in cannabis samples seized by the US Drug Enforcement Administration was 4% in 1995 [9], the average THC concentration across a broader sample of cannabis preparations in 2017 was found to be 17.1% [20]. Moreover, manufacturers of various cannabis products sold in retail stores today, such as oils, edibles, and wax, may contain over 90% THC [8,21].

While public health interest in cannabis use has grown, there remains an unmet need for research on the potential effects of cannabis use on oral health, particularly as it pertains to younger age groups. Virtually all modes of cannabis consumption involve the oral cavity, with smoking or vaping in some manner comprising the majority of use [22,23,24,25]. It is therefore not unreasonable to expect that dental professionals will increasingly be managing young patients who use cannabis and potentially present with related problems in the oral cavity. Orthodontists in particular may be largely impacted because teenagers and young adults comprise a significant proportion of orthodontic patients [26,27,28]. By the same token, they may be uniquely positioned to affect change on these patients as orthodontic treatments typically involve regular appointments at 4–6-week intervals with total treatment durations ranging from months to years [29], thereby facilitating many opportunities for meaningful provider-patient interaction.

Currently, the literature on oral health outcomes associated with cannabis use is modest. Most studies have focused on periodontal disease and found cannabis use to be associated with poorer periodontal health [30,31,32,33,34]. Other studies have explored cannabis use and other oral health outcomes such as caries [35,36,37], soft tissue oral lesions [38,39,40,41], and salivary changes [42,43], though findings were inconsistent, confounded by tobacco use, and limited by sample size. To our knowledge, no studies have yet focused solely on adolescents and young adults.

Given the extent to which prevalence of cannabis use has risen in the US and the potential harm that may follow for younger users, studies investigating its potential effects on oral health are warranted. Our cross-sectional study seeks to preliminarily explore potential associations between cannabis use and several oral health measures and related behaviors among an orthodontic age group (12–25 years of age). Where appropriate, findings will be discussed with regard to orthodontic care, given that orthodontists commonly treat and interact with this age group on a near-monthly basis over an extended period of time.

## 2. Materials and Methods

### 2.1. Data Source

The de-identified data for this study were extracted from the electronic health records (EHRs) of patients that presented to general dental clinics at the New York University (NYU) College of Dentistry between January 2015 and December 2021. This secondary analysis of de-identified data was exempt from review by the NYU College of Dentistry Institutional Review Board.

### 2.2. Analytical Sample

The analytical sample comprised dental patients between the ages 12 and 25 (N = 14,657) who had available charts from the EHR. EHRs from 2015–2021 were included in this study. Patient charts are completed by dental providers chairside during appointments and are approved by attending faculty and the end of the visit. We extracted information regarding cannabis use self-reported by patients as well as dental history and findings, participant age, sex, and lifetime tobacco and alcohol use.

### 2.3. Cannabis Use

With regard to querying cannabis use, during initial and periodic dental visits, patients are asked about their history of recreational drug use based on the following close-ended question: “Do you or did you ever use any recreational drug?” Those who respond yes (vs. no) are subsequently asked to “describe the drug and frequency of use” in an open-ended manner. Responses are inputted into a text-box field chairside.

Two independent raters (authors) individually reviewed each of the qualitative (text) responses during the recoding phase in order to determine affirmative responses regarding cannabis use. Raters accounted for misspellings or alternative names for cannabis (e.g., marijuana, weed, hash), and differentiated current use from lifetime use. Previous studies have adopted timeframes ranging from past 1–3 months in defining current cannabis use [44,45,46]. However, owing to the open-ended nature of the question in this study, only responses that alluded to ongoing cannabis use as of the date appointment were recoded as current use. Lifetime use was defined as having ever used cannabis. We believe this to be a conservative approach that may mitigate potential overestimation of current use. While chronic use would have been the ideal variable for cannabis use, the open-ended nature of the drug use question did not allow for the systematic stratification of cannabis use based on frequency of use. Therefore, both current use and lifetime were adopted as the primary independent variables in this study. After independently reviewing the responses, the raters reconciled discordant responses until 100% inter-rater agreement was achieved.

### 2.4. Oral Health Measures and Behaviors

The oral health outcomes included in this study were limited to those that could be extracted from the EHR and coded in an automatic manner (as opposed to those requiring manual review). Findings of oral lesions, defined in this study as blisters, sores, or ulcers present in the oral cavity, were extracted from social history forms. During visits, patients are asked, “Do you have a history of oral lesions such as blisters, sores, or ulcers?”, and responses (yes versus no) are recorded under their social history.

Clenching and grinding was used as a measure for bruxism. During comprehensive and periodic visits, patients are asked “Do you have a habit of clenching or grinding your teeth?” We also included additional measures relating to the physical integrity of tooth structure, including the presence of wear facets, broken tooth structure, cracked tooth, or fractured restorations. Wear facets are determined during physical head and neck examinations, while the latter outcomes are documented as dental findings.

Clinical attachment loss was the primary indicator of periodontal disease and is calculated by dental providers after periodontal probing. We recoded attachment loss into a dichotomous variable using a cutoff of ≥3 sites with at least 4 mm of attachment loss. This decision was premised upon categorical definitions adopted by the Centers for Disease Control and Prevention (CDC) and the American Academy of Periodontology (AAP) for surveillance of periodontitis, which defines moderate periodontitis as two or more interproximal sites with ≥4 mm (not on the same tooth) or two or more interproximal sites with probing depths ≥5 mm (not on the same tooth) [47]. We were unable to follow the CDC/AAP definitions verbatim due to the lack of equivalently detailed periodontal charting data. Records with missing attachment loss values were excluded from the analysis. Our study included an additional measure of periodontal status based upon whether a patient was classified by their provider as being at high periodontal risk. The criteria for high periodontal risk at NYU College of Dentistry include patients of any age if they have active disease indicators, and patients ≥35 years of age who use tobacco and/or have diabetes or have at least two other known risk factors. Finally, requiring at least one quadrant of scaling and root planning (SRP) was also included as a measure of periodontal disease.

Positive caries status was obtained directly from the EHR if any site of active untreated caries was recorded. Caries is diagnosed based on clinical examination and radiographic evaluations at comprehensive and periodic examinations.

Finally, although not an oral health outcome per se, frequent consumption of sugary beverages or snacks was included as a relevant oral health-related behavior, owing to the potential impact that sugar consumption may have on caries [48] and periodontal status [49,50]. Frequent sugary consumption was based upon the EHR question “Do you frequently have sugary beverages or snacks?”, which patients are asked as a standard part of their social history interview.

### 2.5. Statistical Analyses

First, we calculated the prevalence of self-reported cannabis use and of each condition examined. Using chi-square, we then compared the prevalence of each condition according to lifetime cannabis use. Next, for each comparison, we examined cannabis use in relation to each separate condition again, but in a multivariable manner. Specifically, we entered the indicator for cannabis use as the independent variable for each model with a single dental outcome as the dependent variable, controlling for year, age, sex, and self-reported alcohol and tobacco use. These models were conducted using generalized linear model using Poisson and log link, and the cannabis use variable was associated with adjusted prevalence ratios (aPRs) in each of these separate models. Bivariable and multivariable models were then repeated for current cannabis use.

## 3. Results

Sample characteristics are presented in Table 1. The majority of patients were female (54%), and the mean age was 20 (SD = 4.1). In total, 4.8% and 2.5% of the sample self-reported lifetime and current cannabis use, respectively. Table 2 presents bivariable and multivariable associations between lifetime cannabis use and each outcome. In bivariable tests, those reporting lifetime cannabis use were more likely to have oral lesions and bruxism and were more likely to frequently consume sugary beverages and snacks (ps < 0.05). Table 3 presents bivariable and multivariable associations between current cannabis use and each outcome. In bivariable tests, those reporting current cannabis use were more likely to have oral lesions, bruxism, to be at high periodontal risk, to have SRP completed as part of their dental treatment, and to frequently consume sugary beverages and snacks (ps < 0.05).

With respect to results from the multivariable model, when controlling for age, sex, tobacco and alcohol use, reporting lifetime cannabis use was associated with higher risk of having oral lesions (aPR = 1.41, 95% CI: 1.07–1.85), bruxism (aPR = 1.31, 95% CI: 1.09–1.58), and frequent consumption of sugary beverages and snacks (aPR = 1.27, 95% CI: 1.12–1.41). With respect to current cannabis use, significance was lost for bruxism, but significance was retained for oral lesions (aPR = 1.45, 95% CI: 1.03–2.06) and frequent consumption of sugary beverages and snacks (aPR = 1.26, 95% CI: 1.07–1.48).

## 4. Discussion

Cannabis use remains highly prevalent in the US. Recently, youth cannabis use in particular has garnered public attention owing to favorable legality of recreational use across states, the increase in availability of potent cannabis-containing products, and the widespread media coverage of adverse health effects affecting young users [6,8,9]. Our study sought to preliminarily explore potential associations between cannabis use (both lifetime and current use) and measures of oral health and related behaviors among individuals aged 12–25. Where appropriate, findings are discussed with regard to orthodontic care, given that orthodontists commonly treat and interact with this age group on a near-monthly basis over an extended period of time.

Our findings demonstrate several potentially important associations between cannabis use and oral health, though a low prevalence of reported use (both current and lifetime) is a noted limitation. Firstly, patients who reported either current use or lifetime use of cannabis were more likely to report a history of oral lesions, defined as blisters, sores, or ulcers in the oral cavity, to their provider. Other studies have also suggested that cannabis use increases the risk of developing intraoral soft tissue or mucosal lesions [38]. However, significant heterogeneity exists between studies insofar as study designs and definitions of oral lesions are concerned [51,52], and most studies yielded inconclusive findings [53] or were limited to case reports [40,41,54]. Given our findings, it may be warranted for future studies to further assess the frequency and duration for which these lesions appear, as well as investigate the mode of cannabis consumption. Mucosal lesions such as traumatic ulcerations and erosions are known to be more common in children with appliances (e.g., braces or removeable retainers) than without [55,56], so documenting patient cannabis use and demonstrating additional vigilance is recommended for orthodontists.

In addition, our results show that patients who reported using cannabis in their lifetime were more likely to experience bruxism (adverse clenching and grinding of the teeth). This finding is potentially noteworthy as no previous studies have reported associations between bruxism and cannabis use. To the contrary, anecdotical sources have suggested that use of medical cannabis can alleviate bruxing [57]. The reason for our finding is currently unclear, but it is possible that there may be a confounding factor. For example, clinicians have long suspected psychosocial factors, such as stress, anxiety, and depression, to be predictors of bruxism [58,59,60]. It is possible that the cannabis users in this study—those who seek dental care at a university clinic—may be self-medicating with cannabis in order to cope with stress-inducing psychosocial factors in their lives [61,62,63].

A history of bruxism can have orthodontic implications insofar as the selection of treatment modality is concerned. Specifically, clear aligner therapy is often suggested by orthodontists as the appliance of choice in patients with bruxism since the occlusal coverage of aligners can produce a splint or “night guard” effect and prevent adverse enamel-wear [64]. However, some manufacturers of clear aligners suggest that severe bruxism may be considered a contraindication for clear aligner therapy since the extent of the occlusal forces can deform the plastic aligners and reduce treatment efficacy [65]. Nevertheless, studies supporting this claim are lacking and aligners are routinely changed on a weekly or bi-weekly basis, so it seems unlikely that a patient may structurally compromise their aligners that quickly even in the presence of bruxing.

Our findings also demonstrate that both current and lifetime users of cannabis are more likely to report frequent consumption of sugary beverages and snacks, which could affect their caries or periodontal risk. The dietary habits of cannabis users may foster a carious environment in the oral cavity since THC—the main psychotropic agent in cannabis—is an appetite stimulant that can drive consumption of cariogenic foods [66,67]. Other studies have also found increased consumption of sweet beverages among users [36]. While no association between cannabis use and caries or periodontal status were noted in this study, such knowledge may be of value to orthodontists because the use of common fixed appliances for orthodontic treatment, such as braces, are known to significantly increase caries and periodontal risk owing to the greater difficulty in maintaining adequate oral hygiene [68,69,70]. As such, orthodontists should demonstrate even greater prudence in monitoring the oral hygiene of cannabis-using patients. Moreover, the importance of documenting cannabis use during the initial medical review is again underscored.

While our findings did not demonstrate any significant associations between cannabis use and caries or periodontal disease, we would urge caution in prematurely concluding that no such associations exist. Indeed, an incidental finding in this study is that the prevalence of reported cannabis use (both lifetime and current) appears to be underestimated in this sample when compared to nationally representative samples of the same age range [45,71]. In fact, a recent study has demonstrated that the prevalence of current and lifetime use of several drugs, including cannabis, were markedly underestimated when derived from dental EHRs compared to data from the National Survey on Drug Use and Health [72]. While there are likely several patient-related and provider-related reasons for potential underreporting of use, the authors concluded by encouraging dental professionals to regularly ask patients about their drug use history at dental appointments [72]. From a research perspective, underestimated drug use represents a missed opportunity for furthering studies on oral health and drug use, due to the current lack of complete data that encompass both oral health metrics and drug use measures. Therefore, better EHR design and data collection methods are warranted for future research to build upon the present findings.

Routinely underestimated dental data can also have implications from a public health standpoint. Indeed, there remains a sizeable unmet need regarding treatment for individuals that engage in risky drug use behaviors in the US—an estimated 20.4 million Americans aged ≥12 had a substance use disorder in 2019, but only 2.1 million received any form of treatment [71]. Consequently, patient visits to healthcare providers, such as dentists, are increasingly being viewed as alternative opportunities for screening drug use [73,74]. While there have been general calls for dental professionals to demonstrate more diligence towards asking patients about their drug use history [72], orthodontic visits may represent a unique opportunity for providers to engage in discourse with younger patients about their potential drug use. The 12–25 age range is commonly seen by orthodontists, and the nature of typical orthodontic treatment necessitates regular interactions between provider and patient over a prolonged period. This typically facilitates patient-provider rapport in a manner that is different than that of a patient-physician or even patient-dentist. Consequently, orthodontists may represent an underutilized means of preliminary drug use screening among adolescents and young adults.

Finally, from a clinical standpoint, it is important for orthodontists to query patient cannabis use because the drug contains compounds that can influence physiology and affect treatment outcomes. For example, the non-psychoactive cannabidiol (CBD) compound has been shown to reduce inflammation and attenuate bone remodeling [75,76,77,78]. Orthodontic tooth movement is dependent on an inflammatory cascade to induce bone modeling/remodeling and subsequent tooth movement. Therefore, patients who use cannabis may be at risk for impaired tooth movement and longer treatment durations, the latter having been implicated in root resorption, bone loss, and increased risk of enamel demineralization [79,80]. Further studies in this area would be welcome, and better querying of drug use would only serve to increase the quality of data and facilitate research in this area.

## 5. Limitations

As mentioned earlier, the prevalence of cannabis use was underestimated in this study, perhaps in part because patients are more likely to underreport drug use in healthcare settings [81,82,83] and when questions are open-ended [84]. Reasons for underreporting could be attributable to stigma surrounding drug use [83,85] and the fact that cannabis use was not legalized in New York until 31 March 2021 [6]. It is also possible that some providers simply preferred not to probe patients about their drug use history or habits [73]. We therefore limited the discussion of our findings to the significant differences between study groups and differences in risk. Furthermore, our findings are limited insofar as frequency and mode of cannabis use were not captured. Finally, due to limited records, our study was unable to broaden the definition of oral lesions to include more concerning lesions, such as leukoplakias, and did not control for positive medical history such as diabetes.

## 6. Conclusions

Cannabis users of ages 12–25 are at increased risk for bruxism, oral lesions, and frequent consumption of sugary beverages and snacks. Orthodontists should probe for drug use before commencing treatment and at regular intervals during treatment. The prevalence of cannabis use appears to be greatly underestimated and future research is warranted before ruling out other potential associations between cannabis use and oral health outcomes in this age group. Incidentally, dentists must demonstrate more diligence towards completing EHR forms and probing drug use during review of medical history.

## Figures and Tables

**Table 1:** Sample Characteristics (*n* = 14,657).

	%	*n*
Age, Mean, SD	Mean = 20.0	SD = 4.1
Sex		
Male	46.0	6726
Female	54.0	7903
Year Assessed		
2015	6.3	921
2016	8.7	1278
2017	11.8	1735
2018	14.4	2114
2019	21.7	3176
2020	12.2	1758
2021	24.9	3648
Cannabis		
Lifetime Use	4.8	699
Current Use	2.5	360
Never Used	95.2	13,958
Tobacco		
Lifetime Use	6.5	957
Never Used	93.5	13,700
Alcohol		
Lifetime Use	12.3	1807
Never Used	87.7	12,850

Note. SD = standard deviation.

**Table 2:** Associations between self-reported lifetime cannabis use and oral health outcomes.

	Bivariable Tests			Multivariable Model
Oral Health Measures/Behaviors	Full Sample %	No Use %	Use %	aPR (95% CI)
≥3 sites with ≥4 mm attachment loss	39.3	39.2	40.5	0.93 (0.75, 1.16)
High Periodontal Risk	0.7	0.6	1.1	1.14 (0.53, 2.45)
Scaling and Root Planing Required	4.2	4.2	5.0	0.86 (0.60, 1.23)
Caries	30.8	31.3	21.6	1.07 (0.89, 1.27)
Oral Lesions	4.3	4.0	9.6 ^b^	1.41 (1.07, 1.85) ^a^
Bruxism	9.7	9.1	20.5 ^b^	1.31 (1.09, 1.58) ^b^
Wear Facets	1.6	1.5	2.43	0.92 (0.54, 1.755)
Broken Tooth Structure	1.4	1.4	1.7	1.63 (0.86, 3.10)
Fractured Restoration	1.0	1.0	0.4	0.43 (0.13, 1.39)
Frequent Sugary Beverage/Snack Consumption	26.8	25.9	45.4 ^b^	1.27 (1.12, 1.43) ^b^

Note. aPR = adjusted prevalence ratio. CI = confidence interval. The multivariable model column presents aPRs of cannabis use (as an independent variable) in relation to each separate oral health outcome (represented in each row), controlling for survey year, age, sex, and alcohol and tobacco use. Percentages are column percentages. ^a^ *p* < 0.05, ^b^ *p* < 0.001.

**Table 3:** Associations between self-reported current cannabis use and oral health outcomes.

	Bivariable Tests			Multivariable Model
Oral Health Measures/Behaviors	Full Sample %	No Use %	Use %	aPR (95% CI)
≥3 sites with ≥4 mm attachment loss	39.3	39.1	42.7	0.98 (0.73, 1.31)
High Periodontal Risk	0.7	0.6	1.7 ^a^	1.65 (0.70, 3.93)
Scaling and Root Planing Required	4.2	4.2	6.4 ^a^	0.14 (0.75, 1.76)
Caries	30.8	31.0	22.2	1.09 (0.87, 1.38)
Oral Lesions	4.3	4.1	10.3 ^c^	1.45 (1.03, 2.06) ^a^
Bruxism	9.7	9.5	17.8 ^c^	1.07 0.83, 1.39)
Wear Facets	1.6	1.6	1.9	0.71 (0.33, 1.54)
Broken Tooth Structure	1.4	1.4	1.9	1.78 (0.79, 3.97)
Fractured Restoration	1.0	1.0	0.3	0.29 (0.04, 2.10)
Frequent Sugary Beverage/Snack Consumption	26.8	26.3	46.7 ^c^	1.26 (1.07,1.48) ^b^

Note. aPR = adjusted prevalence ratio. CI = confidence interval. The multivariable model column presents aPRs of cannabis use (as an independent variable) in relation to each separate oral health outcome (represented in each row), controlling for survey year, age, sex, and alcohol and tobacco use. Percentages are column percentages. ^a^ *p* < 0.05, ^b^ *p* < 0.01, ^c^ *p* < 0.001.

## Data Availability

The data that support the findings of this study are available from the corresponding author upon reasonable request.
